# Mini-Flank Supra-12th Rib Incision for Open Partial Nephrectomy for Renal Tumor With RENAL Nephrometry Score ≥10: An Innovation of Traditional Open Surgery

**DOI:** 10.1097/MD.0000000000000692

**Published:** 2015-04-03

**Authors:** Hang Wang, Li-an Sun, Yiwei Wang, Zhuoyi Xiang, Lin Zhou, Jianming Guo, Guomin Wang

**Affiliations:** From the Department of Urology, Zhongshan Hospital, Fudan University, Shanghai, China.

## Abstract

The skill of supra-12th rib mini-flank approach for open partial nephrectomy (MI-OPN) provides an advanced operative method for renal tumor. Compared with laparoscopic and robotic surgery, it may be a feasible selection for the complex renal tumors. We describe our techniques and results of MI-OPN in complex renal tumors with high RENAL nephrometry score (RENAL nephrometry score **≥**10).

Fifty-five patients diagnosed with renal tumors between January 2009 and July 2013 were included in this study. Eligibility criteria comprised of patients with complex renal tumor (RENAL score **≥**10) being candidates for partial nephrectomy (PN). All patients received MI-OPN and all surgeries were performed by a single urologist. The preoperative workup comprised of medical history, physical examination, and routine laboratory tests. Serum creatinine was recorded preoperatively and 2 to 3 months after operation. Operative time, ischemia time, blood loss, operative and postoperative complications, renal function, and pathology parameters were recorded.

MI-OPN was successfully performed in all cases. Mean tumor size was 4.7 cm (range: 2.5–8.1). Mean warm ischemia time was 28.1 minutes (range: 21–39), mean operative time was 105minutes (range: 70–150) and mean estimated blood loss was 68 mL (range: 10–400). Mean postoperative hospital stay was 6.5 days (range: 5–12). Postoperative complications were found in 3 patients (5.5%). The mean pre- and postoperative serum creatinine levels were 76.2 μmol/L (range: 47–132) and 87.1 μmol/L (range: 61–189) with significant difference (*P* = 0.004). The mean pre- and postoperative estimated glomerular filtration rate (eGFR) were 91.5 (range: 34–133) and 82.5 (range: 22–126.5), respectively with significant difference (*P* = 0.024). In an average follow-up of 19.9 months (range: 8–50), no local recurrence or systemic progression occurred.

In conclusion, MI-OPN can combine the benefits of both minimal invasive and traditional open partial nephrectomy (OPN) techniques with a smaller incision. It is an innovation of traditional OPN and suitable for the complex renal tumors with high RENAL nephrometry score safely and effectively.

## INTRODUCTION

The RENAL nephrometry score system consists of radius (tumor size as maximal diameter), exophytic/endophytic properties of the tumor, nearness of tumor deepest portion to the collecting system or sinus, anterior (a)/posterior (p) descriptor, and the location relative to the polar line. Tumor complexity was stratified into three categories: low (4–6), moderate (7–9), and high (10–12) complexity.^1^As one of the standardized surgical approaches of renal tumor, partial nephrectomy (PN) has been widely applied in the last decade, of which indications are being expanded. Laparoscopic partial nephrectomy (LPN) gradually has gained popularity in recent years, which brings a small incision and quick recovery after surgery. Though “zero ischemia” LPN and LPN for central tumors were carried out by some experienced surgeons,^2,3^ LPN is still a technically challenging procedure to most urologists, especially for those with high complexity due to the potential prolonged ischemia time and increased intraoperative hemorrhage.

Over the last decade, there has been great progress in the development of mini-invasive surgery. Laparoscopic surgery and robotic-assisted surgery were carried out worldwide. However, the improvement of traditional open surgery which is not less than mini-invasive surgery tends to be ignored to some extent. The technique of mini-incision for open partial nephrectomy (Mi-OPN) is just one example. The skill has been established by MSKCC in 2006 and improved by our group on the basis of traditional open partial nephrectomy (OPN) surgery.^4,5^ This approach can couple the benefits of both LPN and OPN techniques, providing equivalent oncologic outcomes compared with traditional OPN and LPN. At the same time MI-OPN provided a smaller incision and less estimated blood loss than traditional OPN did and a shorter intraoperative ischemia time and operation time than LPN did.^5^ MI-OPN was applied in peripheral renal tumors at first and then extended in managing complex renal lesions (RENAL nephrometry score **≥**10) after gaining substantial expertise in this procedure from 2009 in our department.

## MATERIALS AND METHODS

### Characteristics of Patient

The study protocol was approved by the Institutional Review Board of the Zhongshan Hospital. Fifty-five patients were included in this study, among which were 35 men and 19 women, who were diagnosed with renal tumors between January 2009 and July 2013 at Zhongshan Hospital Fudan University. Data were collected prospectively and entered into an institutional review board–approved database. Eligibility criteria comprised of patients with complex renal tumor (RENAL nephrometry score **≥**10) being candidates for partial nephrectomy. Exclusions were those with medically high risk, such as severe, preexisting cardiopulmonary, cerebrovascular, or hepatorenal dysfunction. No patient was excluded for reasons of technical complexity of the tumor.

Among all patients, 41 were incidental cases discovered by routine health examinations; 5 came to the hospital complaining of flank soreness, 2 were diagnosed after hematuria, and the other 7 were discovered in the process of treating other diseases. All patients were diagnosed preoperatively with renal neoplastic lesions by ultrasound and computed tomography (CT).

### Preoperative Workup

The preoperative workup comprised of medical history, physical examination, and routine laboratory tests. Serum creatinine was recorded preoperatively and 2 to 3 months after operation. Estimated glomerular filtration rate (eGFR) was calculated using the modification of diet in renal disease (MDRD) formula. A specific abdominal CT with or without magnetic resonance imaging scan was performed to delineate details about tumor location, depth, and proximity to collecting system; the arterial and venous phases were used to evaluate extrarenal hilar renal arterial and venous anatomy. Urine leak was defined as continued Jackson–Pratt drain output for 1 week with a creatinine level of drainage more than twice that of serum creatinine. Preoperative imaging RENAL nephrometry scoring system was applied to help stratification. All cases were of high complexity (**≥**10) in the RENAL nephrometry scoring system.

### Surgical Procedures

**FIGURE 1:**
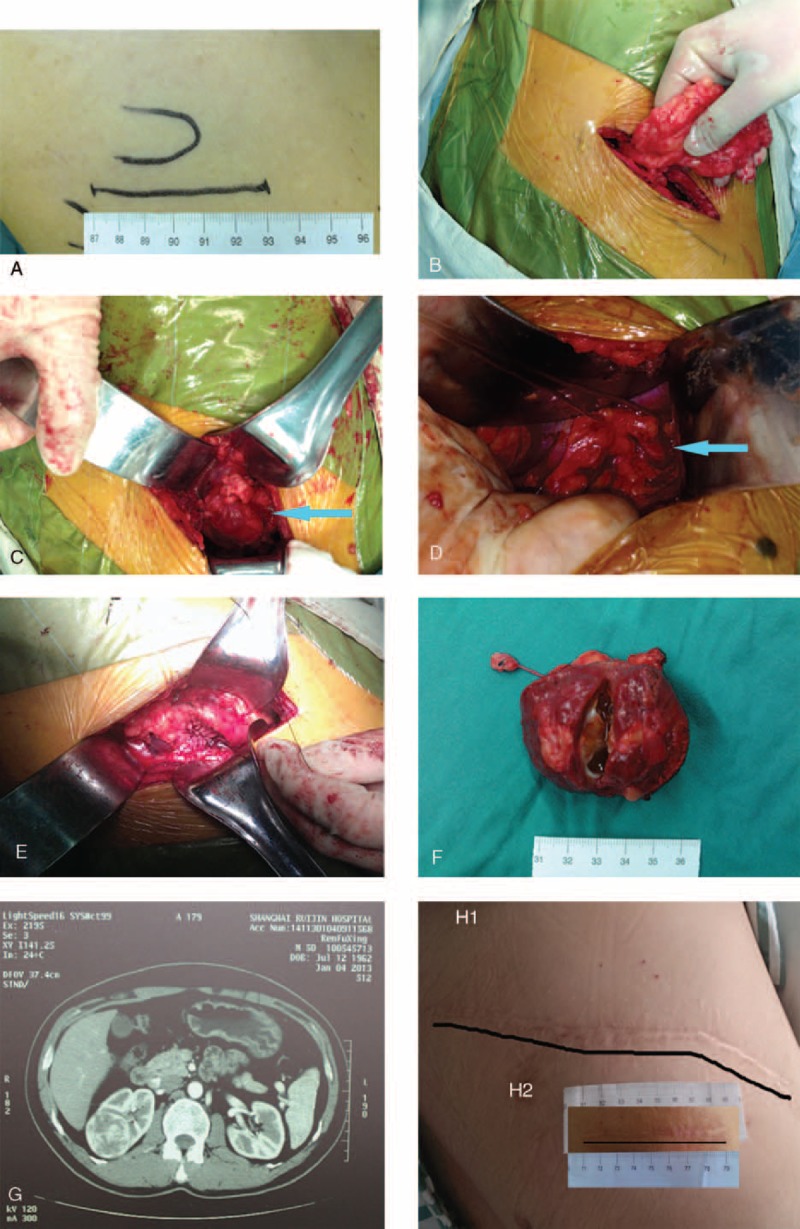
Surgical procedure of Mi-OPN. (A) Mark the intercostal incision above the 12th rib ranged from 7 to 8 cm. (B) Remove the retroperitoneal fat surrounding the Gerota fascia. (C) Perinephric fat surrounding the tumor was dissected and provides a minimal 2- to 3-cm margin by surrounding normal parenchyma in malignant case. The blue arrow points to the neoplasm. (D) Renal artery was isolated and marked by vessel loops. The blue arrow points to the renal artery. (E) All transected blood vessels on the renal incisal surface were clamped with forceps and sutured with 3-0 Vicryl sutures. (F) The specimen consisted of the tumor circumscribed by a rim of normal-appearing parenchyma. (G) The depth and proximity of the tumor to the major renal vessels and collecting system should be carefully estimated preoperatively based on the CT images. (H) The comparison between large flank incision of traditional OPN (h-1) and mini-flank incision of MI-OPN (h-2).

All patients received MI-OPN which was performed by Dr Wang. Intercostal incision above the 12th rib ranged from 7 to 10 cm (Figure 1A,H). The latissimus dorsi, external oblique and internal oblique muscles were transected and the transversus abdominis was divided in the direction of its fibers while preserving the intercostal neurovascular bundle. The transversus abdominis and transversalis fascia near the distal tip of the 12th rib were divided carefully to avoid violating the pleural cavity by displacing the pleura away with the finger by blunt dissection. Then, the retroperitoneal fat surrounding the Gerota fascia should be removed (Figure 1B) carefully and the kidney would be exposed after dissecting the Gerota fascia. Perinephric fat surrounding the tumor was then dissected to provide a clear view of the tumor and a minimal 2- to 3-cm margin by surrounding normal parenchyma in malignant cases was required (Figure 1C). The renal pedicle could be touched with finger to locate the position of renal artery, which was isolated with sharp and blunt dissection and marked by vessel loops (Figure 1D). Then slush ice cooling of the kidney was performed. After the renal artery was clamped with endoscopic bulldog clamp, the renal tumor was sharply incised, leaving several millimeters of grossly normal parenchyma around the tumor with forceps clamping. The depth and proximity of the tumor to the major renal vessels and collecting system should be carefully estimated preoperatively based on the CT images (Figure 1G). If multifocal tumors were revealed by preoperative CT scan, intraoperative ultrasound should be used to identify the suspicious satellite tumors. All transected blood vessels on the renal incisal surface clamped with forceps were sutured with 3–0 Vicryl sutures (Figure 1E), and the collecting system was continuously closed with 4–0 Vicryl sutures. After carefully checking that there was no significant bleeding, 2–0 Vicryl-sutures were used to close the renal parenchyma. The endoscopic bulldog clamps were then removed. The specimen consisted of the tumor circumscribed by a rim of normal-appearing parenchyma and abundant perinephric soft tissue overlying the lesion (Figure 1F). However, perinephric fat was preserved in the benign cases.

## RESULTS

**FIGURE 2:**
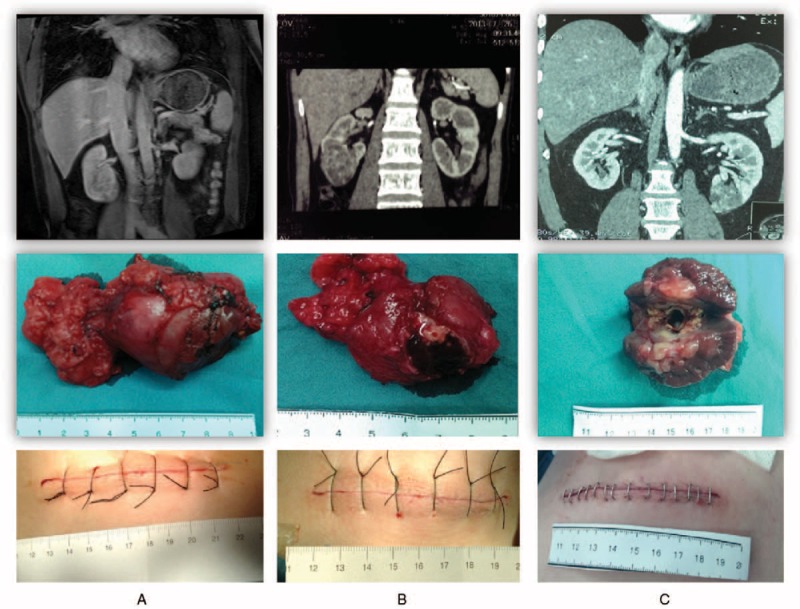
Different cases with high RENAL nephrometry score. (A) RENAL nephrometry score = 12. (B) RENAL nephrometry score = 11. (C) RENAL nephrometry score = 10.

MI-OPN surgeries were successfully completed in all 55 cases. Age of patients ranged from 26 to 75 yd with an average of 52.9 years. Demographic and clinical characteristics of patients were shown in Table 1 and surgical features of these patients were shown in Table 2. Mean warm ischemia time was 28.1 minutes (range: 21–39). Mean tumor size was 4.7 cm (range: 2.5–8.1), mean operative time was 105 minutes (range:70–150), and mean estimated blood loss was 68 mL (range: 10–400). Pelvicalyceal entry repair was performed in 50 patients (90.1%). No patient received intraoperative or postoperative blood transfusion. No intraoperative complication occurred. Mean postoperative hospital stay was 6.5 days (range:5–12). Incision with fat liquefaction occurred on 2 patients and local effusion was found in 1 patient (5.5%). No severe complication was observed, including delayed renal hemorrhage or urinary leakage. Tumors of 50 patients (90.1%) were confirmed as renal cell carcinoma by histopathology and the other 5 cases were angiomyolipoma. Negative surgical margins were confirmed by frozen section analysis for all malignant cases in the operation. The mean pre- and postoperative serum creatinine levels were 76.2 μmol/L (range: 47–132) and 87.1 μmol/L (range: 61–189) with significant difference (*P* = 0.004). The mean pre- and postoperative eGFR were 91.5 (range: 34–133) and 82.5 (range: 22–126.5), respectively, with significant difference (*P* = 0.024) too. The RENAL nephrometry scores of all patients were above 9 (Figure 2A,B,C).

**TABLE 1:**
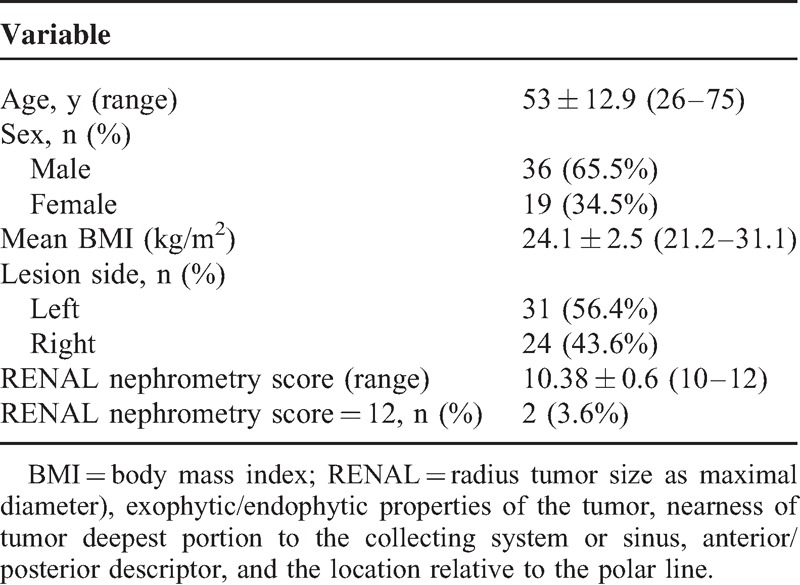
Demographic and Clinical Characteristics of Patients

**TABLE 2:**
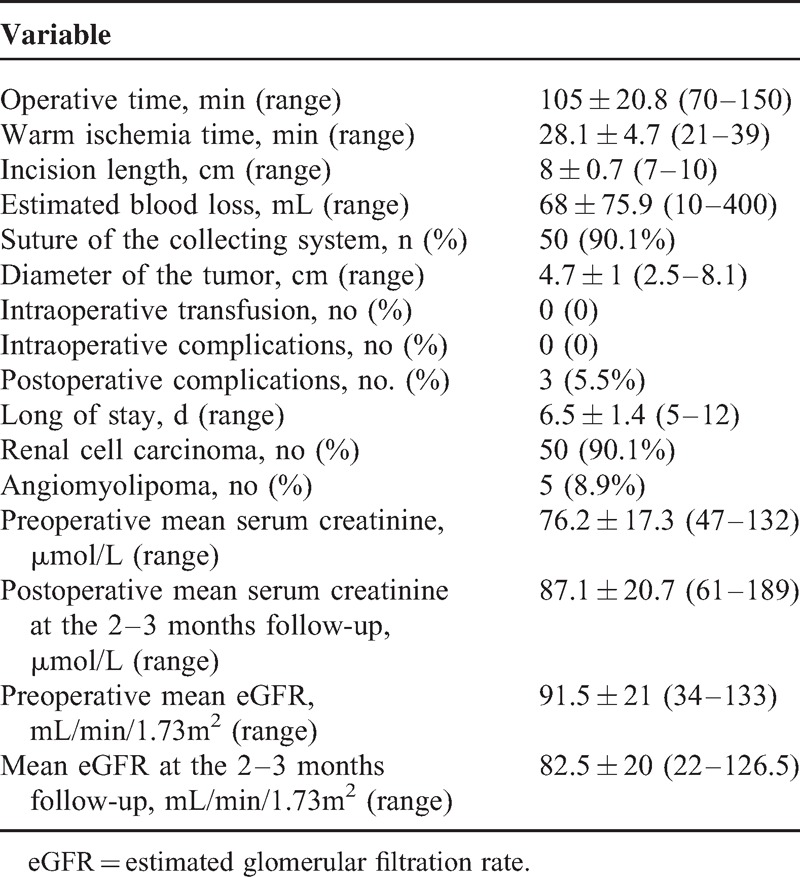
Surgical Features of MI-OPN Surgery

Mean follow-up time for all 55 patients was 19.9 ± 9 months (8–50 months). No recurrence, metastasis, or renal dysfunction was observed.

## DISCUSSION

The proportion of PN is gradually increasing in the treatment for renal tumors.^6^ OPN, LPN, and robot-assisted LPN are now being generally carried out worldwide, which provide similar long-term oncological outcomes in the therapy of T1 renal cancer.^7^

RENAL nephrometry scoring system is reported to be closely associated with the incidence of complication after PN and plays an important role in treatment planning for renal tumor and in assessing the difficulty and risk of the surgical approach.^8–10^ It was reported that RENAL nephrometry scoring system helps in deciding whether to choose OPN or LPN. Generally, OPN is usually performed in moderate (7–9) and high (10–12) complexity renal tumors; however, LPN is mostly applied in low (4–6) complexity cases.^9,10^

OPN through a flank incision has been the preferred approach to renal cancer for many surgeons, and it is still a safe and effective technique of treatment for renal masses,^11,12^ especially in complicated ones. Although providing excellent exposure of the kidney with minimal disturbance of abdominal viscera, the traditional OPN utilizes a large flank incision with or without a resection of the eleventh rib contributing to a significant postoperative pain, a prolonged recovery, or even in many cases an uncomfortable and unsightly flank bulge mainly due to muscle atony, and nerve damage (Figure 1H). Flank approaches can result in an injury to the intercostal nerves with denervation and paresis of the flank musculature, leading to a chronic postoperative pain or flank bulge in 3% to 49% of patients.^13,14^

LPN is increasingly gaining worldwide acceptance as an alternative to open surgery because of its minimal invasion, but it is still considered a major surgical challenge, especially in high RENAL nephrometry scored tumors (10–12). Most guidelines did not adopt LPN as the first choice for small renal tumors,^9^ because even in experienced medical center like Johns Hopkins and Cleveland Clinic, the incidence of complication after LPN is up to 28% and 18%.^15,16^ Moreover, LPN was associated with a significantly longer warm ischemia time while OPN offers shorter operative and ischemia time.^17,18^ Though eGFR at 6-month follow-up did not differ significantly between the two surgical approaches, long-term impact is still hard to be assessed for lack of long-term follow-up.^15,16,19–22^ On the other hand, robot-assisted LPN and LPN are costlier than OPN when including fixed costs.^23^

MI-OPN was developed for renal tumor in our department from 2005 and was applied for managing high-complexity (RENAL nephrometry score >9) renal tumors from 2009.^4^ This approach fully replicates surgical procedures of traditional OPN and provides excellent exposure of the kidney and retroperitoneum with an approximately 8 cm incision (Figure 1H). The oncological outcomes of these cases were satisfactory with a media follow-up time about 19 months. This procedure avoids severe complication, brings smaller incision than traditional OPN does, and provides shorter intraoperative ischemia time than LPN does. Small incision contributed to less complaints of a flank bulge persisting more than 1 year after surgery.^4^ Furthermore, the small incision can be used to retrieve the specimen. A total of 55 patients with high complex renal tumors (RENAL nephrometry score **≥**10) in our study were performed successfully by MI-OPN with no intraoperative transfusion and severe intraoperative complications. The renal function of these cases was affected after operation because of the extensive resection of renal parenchyma.

We recognize the shortcomings of this study are retrospective and nonrandomized. We expanded the indications of mini-flank NSS to high complexity (RENAL nephrometry score **≥**10) after gaining substantial expertise in this procedure for peripheral tumors, which led to an inherent selection bias that could not be overcome.

## CONCLUSIONS

The approach of MI-OPN for PN can combine the benefits of minimal invasive and traditional OPN techniques with a smaller incision. It is an innovation of traditional OPN and suitable for the complex renal tumors with high RENAL nephrometry score.
