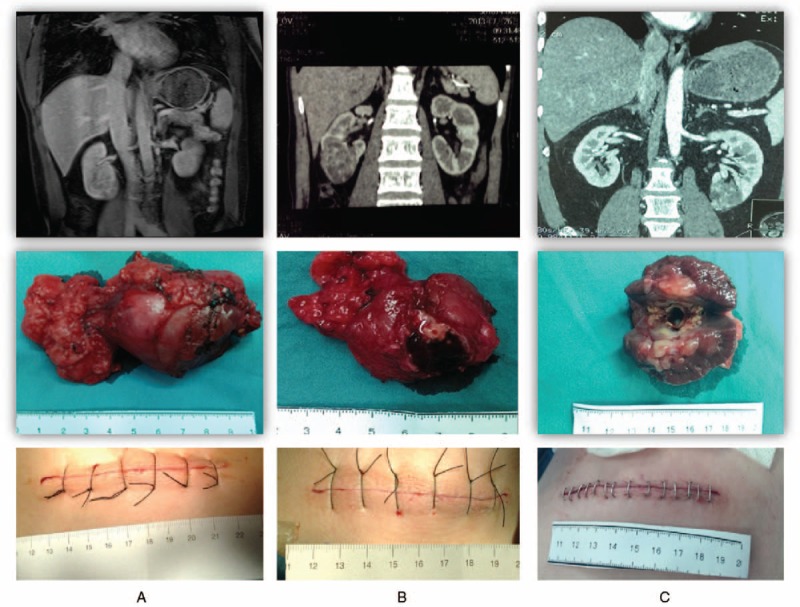# Mini-Flank Supra-12th Rib Incision for Open Partial Nephrectomy for Renal Tumor With RENAL Nephrometry Score ≥10: An Innovation of Traditional Open Surgery: Erratum

**DOI:** 10.1097/01.md.0000465076.56937.bc

**Published:** 2015-05-01

In the article “Mini-Flank Supra-12th Rib Incision for Open Partial Nephrectomy for Renal Tumor With RENAL Nephrometry Score ≥10: An Innovation of Traditional Open Surgery”,^1^ which appeared in Volume 94, Issue 13 of *Medicine*, the middle right panel of Figure 2 was incorrect. The correct figure is given below. The article has since been corrected online.

**Figure:**